# Validity of trunk acceleration measurement with a chest-worn monitor for assessment of physical activity intensity

**DOI:** 10.1186/s13102-022-00492-4

**Published:** 2022-06-10

**Authors:** Masahiko Mukaino, Takayuki Ogasawara, Hirotaka Matsuura, Yasushi Aoshima, Takuya Suzuki, Shotaro Furuzawa, Masumi Yamaguchi, Hiroshi Nakashima, Eiichi Saitoh, Shingo Tsukada, Yohei Otaka

**Affiliations:** 1 Department of Rehabilitation Medicine I, School of Medicine, Fujita Health University, Toyoake, Aichi Japan; 2 NTT Basic Research Laboratories and Bio-Medical Informatics Research Center, NTT Corporation, Atsugi, Kanagawa Japan; 3 Department of Rehabilitation Medicine, Nippon Medical School Chiba Hokuso Hospital, Inzai, Chiba Japan; 4 Department of Rehabilitation, Fujita Health University Hospital, Toyoake, Aichi Japan

**Keywords:** Smart clothing system, Acceleration, Physical activity

## Abstract

**Background:**

Recent advancements in wearable technology have enabled easy measurement of daily activities, potentially applicable in rehabilitation practice for various purposes such as maintaining and increasing patients’ activity levels. In this study, we aimed to examine the validity of trunk acceleration measurement using a chest monitor embedded in a smart clothing system (‘*hitoe’* system), an emerging wearable system, in assessing the physical activity in an experimental setting with healthy subjects (Study 1) and in a clinical setting with post-stroke patients (Study 2).

**Methods:**

Study 1 involved the participation of 14 healthy individuals. The trunk acceleration, heart rate (HR), and oxygen consumption were simultaneously measured during treadmill testing with a Bruce protocol. Trunk acceleration and HR were measured using the "hitoe" system, a smart clothing system with embedded chest sensors. Expiratory gas analysis was performed to measure oxygen consumption. Three parameters, moving average (MA), moving standard deviation (MSD), and moving root mean square (RMS), were calculated from the norm of the trunk acceleration. The relationships between these accelerometer-based parameters and oxygen consumption-based physical activity intensity measured with the percent VO2 reserve (%VO_2_R) were examined. In Study 2, 48 h of simultaneous measurement of trunk acceleration and heart rate-based physical activity intensity in terms of percent heart rate reserve (%HRR) was conducted with the "hitoe" system in 136 post-stroke patients.

**Results:**

The values of MA, MSD, RMS, and %VO_2_R were significantly different between levels 1, 2, 3, and 4 in the Bruce protocol (*P* < 0.01). The average coefficients of determination for individual regression for %VO_2_R versus MA, %VO_2_R versus MSD, and %VO_2_R versus RMS were 0.89 ± 0.05, 0.96 ± 0.03, and 0.91 ± 0.05, respectively. Among the parameters examined, MSD showed the best correlation with %VO_2_R, indicating high validity of the parameter for assessing physical activity intensity. The 48-h measurement of MSD and %HRR in post-stroke patients showed significant within-individual correlation (*P* < 0.05) in 131 out of 136 patients (correlation coefficient: 0.60 ± 0.16).

**Conclusions:**

The results support the validity of the MSD calculated from the trunk acceleration measured with a smart clothing system in assessing the physical activity intensity.

*Trial registration*: UMIN000034967. Registered 21 November 2018 (retrospectively registered).

**Supplementary Information:**

The online version contains supplementary material available at 10.1186/s13102-022-00492-4.

## Background

Recently, there have been several reports on activity monitoring using wearable devices, along with the dynamic development in measurement technology. Accelerometry is one of the main measurement modalities used for monitoring physical activity. A number of studies have reported its usefulness in monitoring the movements of individuals using devices, such as pedometers, which are worn on the waist and used for measuring step counts [1, 2] and wrist-worn type accelerometers [3, 4], which are increasingly becoming common these days.

Given the increasing need for rehabilitation clinical practices to quantify daily activities of patients for the purpose of maintaining and increasing their activity levels, the use of an accelerometer would be beneficial in the assessment of the activity quantification of patients in rehabilitation. However, there may still be some difficulties in applying these devices to patients with motor impairments such as post-stroke paresis, which is frequently observed in the rehabilitation practice. For example, patients with paresis or lower limb injuries frequently use handrails or canes, which may interfere with accurate measurements using a wrist-worn accelerometer [5]. Measuring steps with an accelerometer can be inaccurate for patients with neurological disorders [6] due to reasons such as irregular step patterns and low gait speeds.

Therefore, an alternative methodology optimized for activity quantification is required for patients with motor impairment.

Trunk acceleration measurement may be an option. The measurement of trunk movement should be less influenced by the upper and lower limb motions of patients with motor impairment and thus can be effective in quantifying the physical activity of the patients. The measurement of trunk acceleration has been used in activity monitoring in various manners. For example, there are several studies that evaluated gait parameters such as steps and asymmetry with trunk acceleration [7–9]. The chest-mounted accelerometer is also used for activity recognition [10, 11] and fall detection [12]. However, the usability of trunk acceleration with a chest-worn monitor for the quantification of activity is not well investigated.

In addition, a new index for the quantification of the physical activity using accelerometer may be needed; to date, the use of indices such as step counts and device-specific activity counts which are discrete variables that describe count of movement above a certain pre-set threshold of intensity has been common [13, 14]. However, previous studies have shown that the step and device-specific activity counts may underestimate activity in patients with motor impairment, especially in those with slow walking velocity [6, 15–18], possibly because the devices are developed for general use and the algorithms for step detection may be optimized for healthy subjects, and therefore the slow and small movement of patients with motor impairment [19, 20] may be hard to detect with the pre-set threshold. Therefore, the use of continuous variables that are without thresholds and describe the intensity of movement may be more appropriate to describe the amount of activity of patients with motor impairment whose daily life activity is slower and smaller in intensity compared with healthy individuals.

Therefore, in this study, we aimed to investigate a continuous variable based on trunk acceleration that is suitable to describe intensity of physical activity. For this purpose, we conducted two-step experiments; first, we investigated the validity of several indices based on trunk acceleration measurements for the assessment of the intensity of physical activity, in comparison with the exercise intensity determined via expiratory gas analysis and heart rate measurement. Second, we conducted 48-h simultaneous measurements of acceleration and heart rate in post-stroke inpatients, to investigate the feasibility and validity of measurements in rehabilitation clinical practice. A smart clothing system (‘hitoe’ system), which is a wearable monitoring system embedded with chest-worn accelerometer and heart rate monitor, was used for measurement.

## Materials and methods

### Participants

#### Study 1

Fourteen healthy adults (10 males; mean age of 29 ± 5 years) with no medical history of neurological, musculoskeletal, cardiovascular, or respiratory diseases participated in this study (Table 1). Individuals who received medication that could potentially affect performance were excluded.

**Table 1:** Participants' characteristics

Variables	Study 1 (Healthy individuals)	Study 2 (Stroke patients)
Age, years	29 ± 5	66 ± 15
Height, cm	166.3 ± 9.0	160.7 ± 16.9
Weight, kg	58.7 ± 11.0	58.7 ± 14.2
Sex, male/female	10/4	87/49
Diagnosis, intracerebral hemorrhage/cerebral infarct/subarachnoid hemorrhage		59/64/13
Time after stroke, days		31 (4–95)
SIAS motor score (0–25)		14.6 ± 8.6
Severe (0–10)/moderate (11–20)/mild (21–25)		43/65/28
FIM total score		74.7 ± 33.9
FIM motor score		51.1 ± 25.7
FIM cognitive score		24.0/10.1

#### Study 2

Participants were recruited from the acute- or subacute post-stroke patients who underwent inpatient rehabilitation at Fujita Health University Hospital, Convalescent Rehabilitation Ward, between January 2018 and February 2021. The inclusion criteria were: (1) patients diagnosed with cerebral hemorrhage, cerebral infarct, or subarachnoid hemorrhage, (2) patients admitted in rehabilitation ward within120 days after onset. The exclusion criteria were: (1) presence of orthopedic disease and/or severe cardiopulmonary disease that limits daily living activity, (2) beta-blocker usage, (3) presence of arrhythmia including atrial fibrillation, and (4) unstable medical condition (e.g., deep vein thrombosis, aspiration pneumonia, or superimposed sepsis). A total of 136 patients were included. Demographic variables of the patients are shown in Table 1.

Each subject’s functional motor level was quantified using motor score of Stroke Impairment Assessment Set (SIAS), which scores motor function from 0 to 25 [21]. According to the SIAS, the patients were divided into three groups; severe (SIAS 0–10), moderate (11–20), and mild (21–25). The levels activities of daily living of the patients were shown with Functional Independence Measure (FIM) [22].

This study was complied using the principles of the Declaration of Helsinki and was approved by the Medical Ethics Committee of Fujita Health University. All the participants provided written informed consent prior to participation.

### Procedures

#### Study 1: Measurement

Each participant underwent treadmill testing following Bruce protocol [23]. Respiratory gas analysis during exercise testing was performed using a mobile aerosol monitor (AE-100i, Minato Medical Science, Tokyo, Japan) to measure oxygen consumption (VO_2_). The participants wore a face mask to sample exhaled air and the VO_2_ was continuously measured using breath-by-breath method. The monitor was calibrated before and after each testing session using verified calibration gases.

Participants were asked to avoid any high-intensity exercise and alcohol or caffeine consumption 24 h prior to the assessment. Before these tests, the resting VO_2_ while sitting was measured. According to the Bruce protocol, participants started exercising at level 1 with a treadmill speed of 2.7 km/h and an incline of 10% gradient for 3 min. The speed and inclination were subsequently increased at 3-min periods in the following manner: level 2, 12% incline at 4.0 km/h; level 3, 14% incline at 5.5 km/h; level 4, 16% incline at 6.8 km/h; level 5, 18% incline at 8.1 km/h; level 6, 20% incline at 8.9 km/h; and level 7, 22% incline at 9.7 km/h. We considered VO_2_ to have reached the maximum value if the participants satisfied at least three of the following four criteria: (1) maximum voluntary exhaustion, as measured by the Borg CR-10 scale; (2) presence of a heart rate plateau (ΔHR between two consecutive work rates ≤ 4 beats·min^−1^); (3) presence of a VO_2_ plateau (ΔVO_2_ between two consecutive work rates < 2.1 mL·kg^−1^⋅min^−1^); and (4) maximal respiratory exchange ratio ((RERmax) > 1.1) [24, 25].

**Fig. 1:**
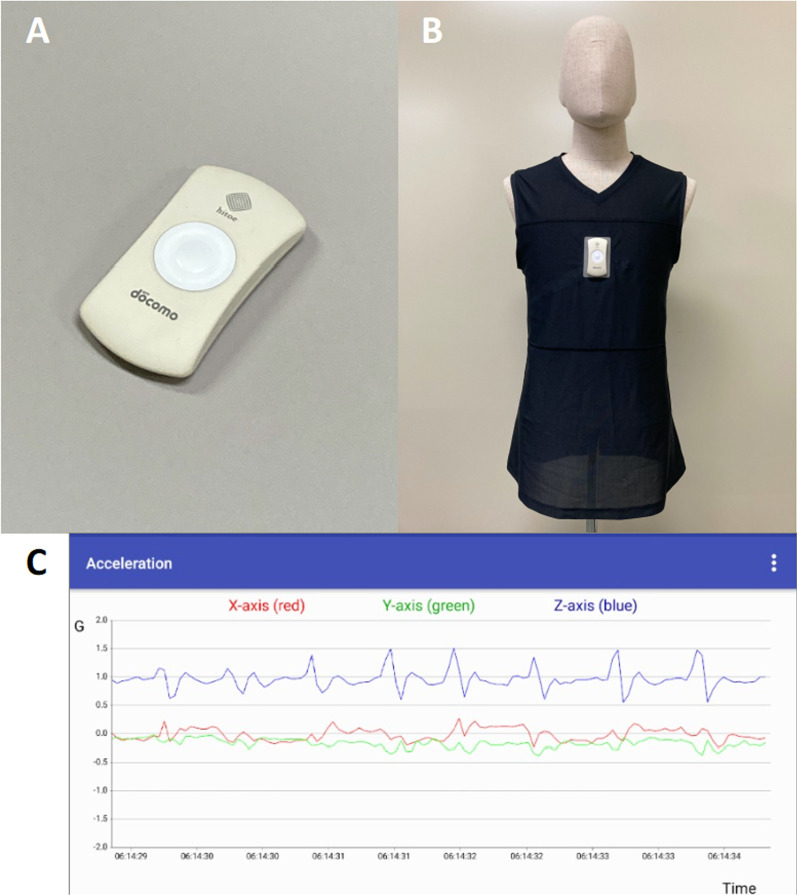
The ‘*hitoe’* transmitter, the ‘*hitoe’* wear, and the smartphone application. The *'hitoe'* transmitter (**A**) is placed on the chest of the *'hitoe'* wear (**B**). The data sent to the smartphone via Bluetooth can be seen on the smartphone application (**C**)

Trunk acceleration and heart rate (HR) was measured using a ‘*hitoe’* smart clothing system (Fig. 1; NTT Corp., Tokyo, Japan and Toray Industries Inc., Tokyo, Japan) [26]. This smart clothing system comprised a ‘*hitoe’* wear, ‘*hitoe’* transmitter, and smartphone application. An accelerometer embedded in the ‘*hitoe’* transmitter placed on the chest measured the trunk acceleration. The HR was measured with the chest electrode embedded in the ‘*hitoe’* wear. The accuracy of heart rate measurement of this system has been previously reported [27]. The sampling rate was 25 Hz. The transmitter sent the data to a smartphone using Bluetooth Low Energy (BLE). The smartphone application was created by authors using the ‘*hitoe’* SDK kit (NTT DOCOMO Inc., Tokyo).

#### Study 1: Parameters

The intensity of the activity was assessed by the percent VO_2_ reserve (%VO_2_R), a gold standard for the assessment of exercise intensity [28].

%VO_2_R was calculated using the following equation:

$$\% VO_{2} R = \left( {VO_{2} - resting\, VO_{2} } \right)/\left( {Maximum\, VO_{2} \,during\, treadmill \,testing - resting\, VO_{2} } \right).$$

The VO_2_ value during sitting and the maximum VO_2_ value during treadmill exercise testing using the Bruce protocol were used for measuring resting and maximum VO_2_, respectively.

In addition, percent HR reserve (%HRR) was calculated from HR measured. %HRR is also used as an index of exercise intensity [29] that strongly correlates with %VO_2_R and is considered equivalent to %VO_2_R [30, 31].

%HRR was calculated as follows:

$$\% HRR = \left( {HR - resting\,HR} \right)/\left( {maximum\,HR \,during\, treadmill\, testing - \left. {resting\,HR} \right\}} \right)$$.

Several movement quantification indices based on trunk acceleration were compared with %VO_2_R in this study. The moving mean, moving standard deviation, and moving root mean square over a window of 50 samples (2 s) of the trunk acceleration were calculated using data for the last 2 s (50 data points). The norm of acceleration was calculated using the following equation:

$$Norm = \sqrt {(x^{2} + y^{2} + z^{2} }$$),

where x, y, and z represent the vertical, lateral, and anterior/posterior axes, respectively.

%VO_2_R and acceleration-based indices used for the analyses were averaged during the middle 1 min of each 3-min stage during the treadmill testing.

#### Study 2: Measurement and Parameters

A 48-h measurement session was conducted using the ‘*hitoe’* smart clothing system for post-stroke inpatients in the convalescent rehabilitation ward. The patients wore a ‘*hitoe’* wear for 48 h consecutively, starting from the morning of the first measurement day. The heart rate and trunk acceleration were measured using a chest-worn accelerometer and a heart rate monitor embedded in ‘*hitoe’* wear. The %HRR and MSD of the acceleration value were calculated as Study 1. The time course of the values averaged for every 30 min were used for analysis.

### Analyses

#### Study 1

The normality assumption was checked using the Shapiro–Wilk test. Pearson's or Spearman's correlations were used to assess the simple relationship between %VO_2_R, %HRR and acceleration-based indices. To investigate whether the acceleration-based indices differentiate the different levels of physical activity defined in the Bruce protocol, the values of MA, MSD, and RMS, and %VO_2_R as a reference, at levels 1 to 4 in the Bruce protocol were compared. The one-way repeated measures analysis of variance (ANOVA) or Friedman test was used for comparison between the levels, and when significant, post-hoc multiple comparisons were performed using Tukey's HSD test (parametric) or Steel–Dwass test (non-parametric).

To investigate within-subject relationships between the acceleration-based indices and %VO_2_R and its variability, three linear regressions were performed for each subject: values of %VO_2_R versus MA, %VO_2_R versus MSD, and %VO_2_R versus RMS. Mean values and standard deviations (SD) for intercepts, slopes, and coefficients of determination were calculated for each regression.

Sample size was calculated on the basis of the previously shown correlation between the acceleration-based indices (RMS and device-specific parameters) and VO_2_ measurements [32, 33], using software G*Power, version 3.1.9.2 [34]. Minimum required sample size was calculated as eight (1-β 0.95, α 0.05). To minimize the effect of data loss, we recruited 14 participants for this study.

#### Study 2

The normality assumption was checked using the Shapiro–Wilk test. Pearson's or Spearman's correlations were used to assess the bivariate relationships between %HRR and MSD. To investigate within-subject relationships between the %HRR and MSD, linear regressions were performed for each subject. Mean value and standard deviation (SD) for intercepts and slopes were calculated. The one-way ANOVA (parametric) or Kruskal–Wallis test (non-parametric) was used for comparison of regression slopes among mild, moderate, and severe paresis groups, and when significant, post-hoc multiple comparisons were performed using Tukey's HSD test (parametric) or Steel–Dwass test (non-parametric).

Statistical analyses of experiment 1 and 2 were performed using JMP11 software (SAS Institute Inc., Cary, NC, USA). The significance level was set at 5%.

This study has been registered with the number of UMIN000034967, on 21/11/2018.

## Results

### Study 1

**Fig. 2:**
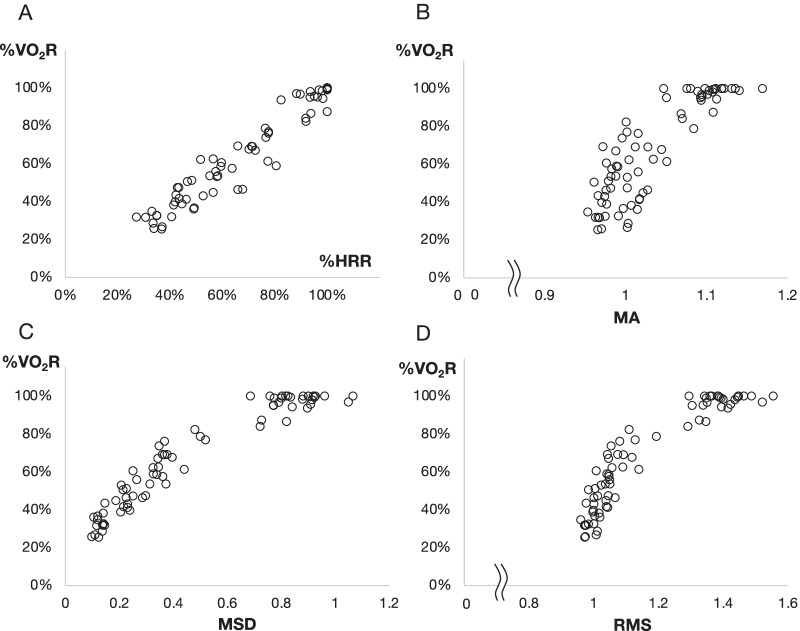
Scatter plot of %VO_2_R versus %HRR, %VO_2_R versus MA, %VO_2_R versus MSD, and %VO_2_R versus RMS. The scatter plots of acceleration indices: %VO_2_R versus %HRR (**A**: r = 0.97, *P* < 0.01), %VO_2_R versus MA (**B**: r = 0.87, *P* < 0.01), %VO_2_R versus MSD (**C**: r = 0.96, *P* < 0.01), and %VO_2_R versus RMS (**D**: r = 0.92, *P* < 0.01)

First, the simple relationship with the Pearson correlation between VO_2_, HR, and acceleration-based indices were assessed. Significant correlations were observed among %VO_2_R and %HRR, %VO_2_R and MA, %VO_2_R and MSD, and %VO_2_R and RMS (r = 0.97 and *P* < 0.01, r = 0.87 and *P* < 0.01, r = 0.96 and *P* < 0.01, r = 0.92 and *P* < 0.01, respectively). The scatter plots are shown in Fig. 2.

To evaluate the strength and the variety of within-individual relationship between the acceleration-based indices and VO_2_R, the mean, SDs and CVs for the intercepts and slopes from the individual linear regression models and their coefficients of determinant were evaluated (Table 2). The averaged coefficients of determination for %VO_2_R versus MA, %VO_2_R versus MSD, and %VO_2_R versus RMS were 0.89 ± 0.05, 0.96 ± 0.03, 0.91 ± 0.05, respectively.

**Table 2:** Intercepts, slopes, and coefficients of determinants from the individual linear regression models

	MA	MSD	RMS
y intercept	− 4.98 ± 2.27	0.27 ± 0.07	− 1.00 ± 0.34
Slope	5.47 ± 2.20	0.86 ± 0.13	1.44 ± 0.29
R^2^	0.90 ± 0.05	0.96 ± 0.03	0.91 ± 0.05

MA, moving average of acceleration; MSD, moving standard deviation of acceleration; RMS, root mean square of acceleration; SD, standard deviation; CV, coefficient of variation

**Fig. 3:**
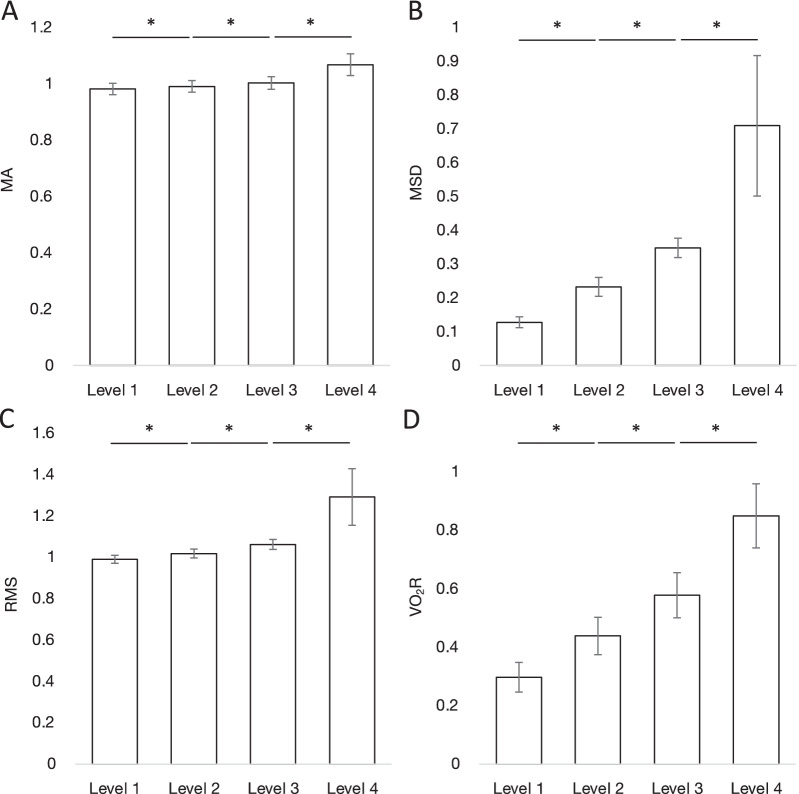
The acceleration indices and VO_2_R values in each level of Bruce protocol. The values of MA (**A**), MSD (**B**), RMS (**C**), and %VO_2_R (**D**) at levels 1 to 4 are shown. *P < 0.01

Then, the discriminative capacity of the acceleration-based indices in detecting different levels of exercise task were tested comparing the data of levels 1, 2, 3, and 4 in Bruce protocol. The values of MA, MSD, RMS, and %VO_2_R at levels 1 to 4 in the Bruce protocol are shown in Fig. 3. The values at level 1–4 were 0.98 ± 0.02, 0.99 ± 0.02, 1.00 ± 0.02, and 1.06 ± 0.04 for MA; 0.13 ± 0.02, 0.23 ± 0.03, 0.34 ± 0.03, and 0.70 ± 0.22 for MSD; 0.99 ± 0.02, 1.01 ± 0.02, 1.06 ± 0.02, and 1.28 ± 0.15 for RMS; and 32.6 ± 4.9%, 47.0 ± 6.2%, 61 ± 7.9%, and 86.5 ± 11.6% for VO_2_R, respectively. Significant differences between levels 1 and 2, levels 2 and 3, and levels 3 and 4 can be observed in all indices (*P* < 0.01).

The correlation between %VO_2_R and acceleration-based indices and %HRR within each level is shown in Table 3. Significant correlations between %VO_2_R and MA, MSD, and RMS were found at level 4, while correlations between %VO_2_R and %HRR were significant at levels 1, 2, and 4. Scatter plots of %VO_2_R and MA, MSD, RMS, and %HRR at levels 1 to 4 are shown in Additional file 1: Fig. S1.

**Table 3:** Correlation coefficient between %VO2R versus %HRR and acceleration indices

%VO_2_R vs	Level 1	Level 2	Level 3	Level 4
%HRR	0.54*	0.56*	0.49	0.62*
MA	0.18	0.01	0.23	0.56*
MSD	0.43	0.33	0.50	0.78**
RMS	0.34	0.27	0.27	0.76**

*P < 0.05, **P < 0.01

### Study 2

**Fig. 4:**
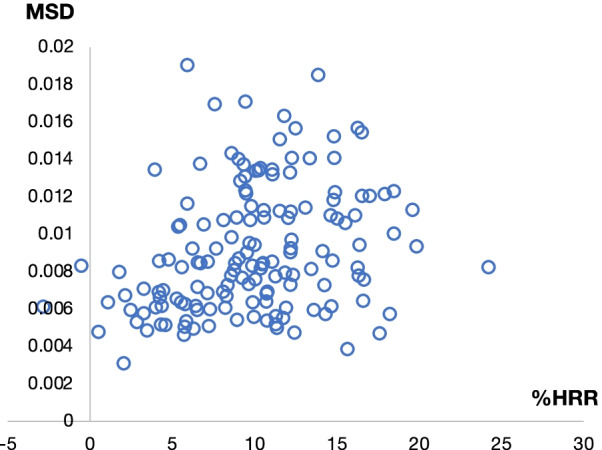
The scatter plot of averages of MSD and %HRR (r = 0.29, *P* < 0.01)

A comparison between %HRR, which was previously reported to be equivalent with the %VO_2_R [30], and MSD, which was best correlated with VO_2_R in the experimental setting, was conducted in real-life setting (48 consecutive hours) of the post-stroke patients. The correlation coefficient between the average of the %HRR and MSD was r = 0.29 (*P* < 0.01, Fig. 4).

The correlation between MSD and %HRR within each participant was also investigated. Significant correlation was observed in 131 out of 136 patients (96.3%). The average of correlation coefficient was 0.60 ± 0.16.

**Fig. 5:**
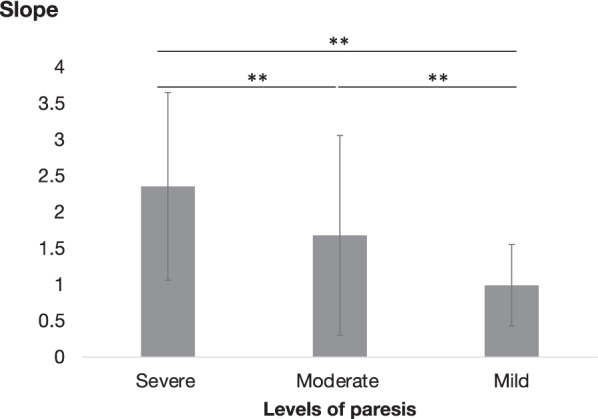
Regression slope (x = MSD, y = %HRR) and levels of paresis. Severe, moderate, and mild groups include patients with SIAS 0–10, 11–20, and 21–25, respectively. * < 0.05, ** < 0.01

To investigate the variety of within-individual relationship between the MSD and %HRR, the mean and SDs for the intercepts and slopes from the individual linear regression models were evaluated. The slopes were 1.75 ± 1.31 and the intercepts were 0.01 ± 0.04. The values of slope in mild, moderate, and severe paresis groups are presented in Fig. 5. Significant differences among patients with severe, moderate, and mild paresis were observed ( 2.36 ± 1.30, 1.68 ± 1.38 and 0.99 ± 0.56, respectively).

## Discussion

In this study, the acceleration-based measurement of physical activity was validated in two steps. First, the relationship between %VO_2_R, %HRR, and the acceleration-based movement indices, MA, MSD, and RMS—calculated from the measurement of trunk acceleration using a smart clothing system—was examined. Overall, the acceleration-based indices were significantly correlated with %VO_2_R, as well as %HRR. The results of the regression analysis of each subject showed that MA, MSD, and RMS all fit the linear regressions, with MSD showing the best fit with the individual linear regressions. Using these acceleration indices, the different levels of exercise intensity defined in the Bruce protocol were clearly identified. Second, 48-h of activity measurements using MSD and %HRR was conducted in 136 post-stroke patients. Although the group analysis only shows weak correlation between averaged daily MSD and %HRR, significant correlation between the values of MSD and %HRR within individual measurements were observed in 96.3% of the patients. The slopes of the individual regression were significantly different between different levels of paresis.

The overall correlation between the trunk acceleration with waist-worn accelerometer and the oxygen consumption has been shown previously [32, 33]. The results of this study showed that this correlation is also seen between the values measured by chest-worn accelerometer and the exercise intensity estimated from the oxygen consumption that is frequently used in the exercise prescription in the rehabilitation practice [35]. In addition, we tested several indices of acceleration, such as MA and MSD—which are basic indices that represent the amplitude and fluctuation of values—and the RMS—which has been used in previous studies that quantified running using an accelerometer [32, 36]. Among these indices, MSD exhibited the strongest correlation with VO_2_R, and the least variability between the subjects. This may be related to the inclusion of gravitational acceleration. The absolute measurement value of accelerometer includes error from the gravity acceleration, which is difficult to separate from the dynamic component of acceleration [37, 38]. Considering that the measurement of acceleration is affected by environmental conditions such as temperature [39] and that the necessity of frequent calibration would complicate measurement, measurement values that do not include gravitational acceleration could represent a better alternative. While MA and RMS are indices that include gravitational acceleration, MSD is an index of fluctuations from the moving average, which focuses more on the dynamic component of values. Although there may be more sophisticated methodology such as the use of autocalibration methodology to eliminate the gravitational acceleration [40], the simple solution to calculate moving standard deviation without complex data analysis can be easily applied regardless of the measurement devices.

The correlation between the VO_2_R representing relative increase in oxygen consumption and the trunk acceleration is logically derived from the intensity of physical motion of the trunk. In fact, the trunk is the heaviest body segment [41, 42]; thus, its movement can largely affect oxygen consumption. Therefore, trunk movement can possibly provide more accurate measurements on exercise intensity than upper-limb movement, which varies extensively in patients with motor impairment [43]. Although the measurement of trunk movement with a chest-mounted accelerometer may not be as easy as with wrist-worn accelerometers, the use of a smart clothing system can make it more feasible [44, 45].

The acceleration indices also identified different levels of exercise tasks, defined by the speed and inclination in the Bruce protocol. This level identification is reasonable considering that the large stride related to the high treadmill walking speed and inclination requires a large movement of the human body [46], with the cadence and frequency of the steps also increasing to adjust to the high treadmill speed [47]. Among the indices, MSD showed the least overlap in values between the levels 1, 2, 3, and 4, indicating the best accuracy of all in describing the physical intensity of the activity. The low within-level correlation between %VO_2_R and acceleration indices in levels 1, 2, and 3 should indicate that acceleration measurement of the trunk reflects the task itself, irrespective of individual fitness while walking; the speed and inclination determine the acceleration values. In contrast, a high within-level correlation between %VO_2_R and acceleration indices was observed in level 4, in which trunk movement can be more dynamic. These differences should be related to the difference between the acceleration-based indices and the %VO_2_R and %HRR; physical activity intensity as measured by accelerometers reflects the actual physical movements [48], while %HRR and %VO_2_R are based on blood and supplied oxygen [30]. The simultaneous measurement of these two aspects of physical activity may provide a deeper understanding of the activity.

**Fig. 6:**
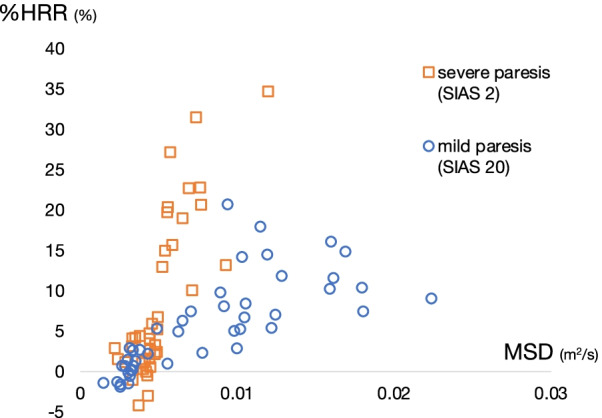
Representative scatter plot of MSD versus %HRR in patients with mild and severe paresis

In the second study, we investigated how these measurements appear in patients with motor impairments. The relationship between the MSD and %HRR, reflecting actual movement and blood provision-based physical intensity, in post-stroke patients was examined. The MSD and %HRR were significantly correlated in 96.3% of the patients, including those with severe paresis. These results indicate that MSD, a continuous variable without pre-set threshold, can be used to measure physical activity in patients with various levels of motor impairment. In addition, the results showed large variability in the relationship between the MSD and %HRR, with the slope of the regression related to the severity of the paresis. This variability in regression slope may reflect the varied level of efficiency of the physical activity in the patients. McGregor et al. reported that the relationship between acceleration measurements and oxygen consumption can vary with exercise experience [32]. Accordingly, it is reasonable that the relationship between supply and output varies extensively in people with motor impairment. For example, a patient with severe paresis may need more blood supply, resulting in an increase in %HRR, while performing less physical movement corresponded to the MSD when compared with a patient with mild paresis (Fig. 6). Evaluating this relationship will expand the possibilities of activity measurement, useful for evaluating the amount of activity and the movement efficiency for each patient.

### Limitations

In this study, the acceleration indices based on trunk acceleration measurement were compared with %VO_2_R using Bruce protocol only in healthy subjects, although this study aimed to develop a trunk acceleration-based indices that can be used for physical activity intensity measurement including patients with motor impairment. Actually, the exercise loading at maximal oxygen consumption is impossible in the patients due to the motor impairment and the same experimental setting cannot be applied. Therefore, in study 2, we tested this with more simplified methodology but with larger samples in clinical setting; the 48-h simultaneous measurement of acceleration and HR was conducted in 136 post-stroke inpatients. The physical activity intensity was measured with %HRR calculated from HR and strongly correlates with %VO_2_R. The simultaneous measurement of acceleration and %HRR showed significant within-individual correlations between MSD and %HRR. Although the %HRR measurement does not directly quantify oxygen consumption, the large sample size may support the certainty of the results. In addition, the second study conducted with the clinical population would support the feasibility of this methodology in clinical measurement of physical activity intensity.

## Conclusions

In this study, we evaluated the acceleration-based measurement of physical activity using a chest-worn accelerometer in two steps. The first study with healthy individuals revealed a high correlation between the trunk acceleration indices, especially MSD, and %VO2R, thus supporting the usability of trunk acceleration measurement using a chest-worn accelerometer for assessing the physical activity intensity. The second study involving post-stroke rehabilitation patients demonstrated the possible advantage of this method in activity monitoring that enables simultaneous measurement of actual activity and blood supply to show activity efficiency. Further exploration of these methodologies may provide a meaningful and clinically viable model for using activity monitoring in rehabilitation settings.

## Supplementary Information

**Additional file 1: Fig. S1**. Scatter plot of %VO2R vs. %HRR, %VO2R vs. MA, %VO2R vs. MSD, and %VO2R vs. RMS at levels 1 to 4 in the Bruce protocol.

## Data Availability

The data collected and analyzed during the current study are available from the corresponding author on reasonable request.

## Abbreviations

VO_2_R: Percent VO_2_ reserve
MA: Mean acceleration
MSD: Mean standard deviation
RMS: Root mean square
