# Supplementary material for: Validity of trunk acceleration measurement with a chest-worn monitor for assessment of physical activity intensity
Source: BMC Sports Sci Med Rehabil. 2022 Jun 10;14:104. doi: 10.1186/s13102-022-00492-4 (PMC9185863; doi:10.1186/s13102-022-00492-4)
Supplement: Supplementary file 1 — Additional file 1: Fig. S1. Scatter plot of %VO2R vs. %HRR, %VO2R vs. MA, %VO2R vs. MSD, and %VO2R vs. RMS at levels 1 to 4 in the Bruce protocol. [file 13102_2022_492_MOESM1_ESM.pdf]

Supplementary figure 1

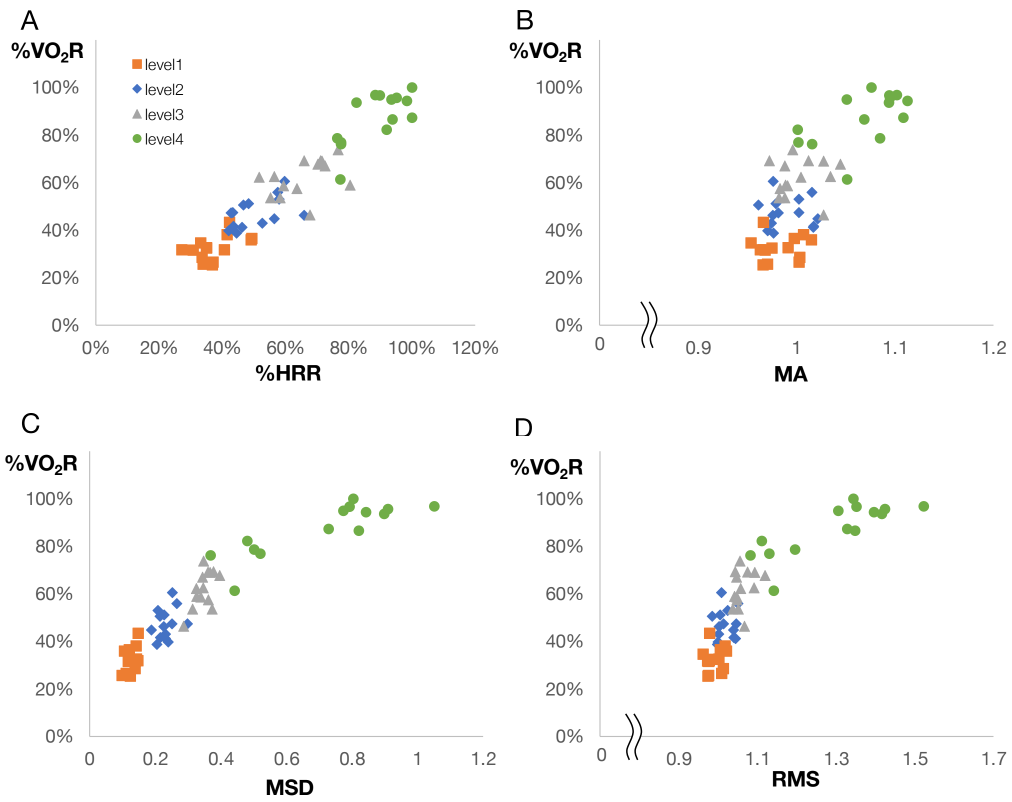

Supplementary figure 1. Scatter plot of %VO2R vs. %HRR (A), %VO2R vs. MA (B), %VO2R vs. MSD (C), and %VO2R vs. RMS (D) in level 1 (orange, square), level2 (blue, diamond), level3 (gray, triangle) and level4(green, circle).
